# Gender differences in Generalized Anxiety Disorder: a cross-sectional multicenter study

**DOI:** 10.1192/j.eurpsy.2026.11954

**Published:** 2026-07-31

**Authors:** M. Buoli, L. M. Affaticati, E. Capuzzi, F. Colmegna, M. Clerici

**Affiliations:** 1 Fondazione IRCCS Ca’ Granda Ospedale Maggiore Policlinico, Milan; 2 University of Milan Bicocca; 3 Fondazione IRCCS San Gerardo dei Tintori, Monza; 4 ASST Bergamo Ovest, Bergamo; 5 University of Milan Bicocca, Milan, Italy

## Abstract

**Introduction:**

Generalized Anxiety Disorder (MDD) is a disabling mental condition, with a prevalence in women twice than men. Despite the implementation of precision and gender medicine, little data are currently available on gender differences in GAD.

**Objectives:**

Purpose of this study is to identify gender differences in clinical features and outcomes of patients affected by GAD to implement individualized treatment strategies.

**Methods:**

A cross-sectional, retrospective study was conducted. Medical records of GAD patients from three mental health services were reviewed, and socio-demographic and clinical variables were extracted. Continuous and qualitative variables were compared according to gender respectively by unpaired t-tests and chi-square tests (with odds ratio-[OR] calculation where appropriate).

**Results:**

The total sample included 243 patients (162 women and 81 men). Female patients compared to males resulted: to be more frequently married or in partnership (61.7% versus 44.4%; χ^2^=8.68, p=0.03), to have less frequently cannabis use disorder before GAD onset (0.6% versus 7.4%; χ^2^=13.71, p=0.01), to have less frequently alcohol use disorder after GAD onset (0.6% versus 6.2%; χ^2^=13.68, p<0.01), to have more frequently committed attempted suicides (4.9% versus 0.0%; χ^2^=4.14, p=0.05), to present more often a history of obstetrical complications (low birth weight or preterm birth) (5.5% versus 0.0%; χ^2^=4.14, p=0.03; OR=1.54).

**Conclusions:**

The results of this study support gender-specific medicine with personalized treatments for males and females with GAD. Males are more likely to engage in substance abuse, while females are more likely to engage in self-harm. Specific psychoeducational interventions should be planned, and pharmacotherapy should be chosen based on their interactions with substances of abuse or their anti-suicidal efficacy. The results of this study need to be confirmed by future research.

**Disclosure of Interest:**

None Declared

